# The Temporal Contribution of the *Gbx2* Lineage to Cerebellar Neurons

**DOI:** 10.3389/fnana.2017.00050

**Published:** 2017-07-21

**Authors:** Nellwyn Hagan, Juliana Guarente, Debra Ellisor, Mark Zervas

**Affiliations:** ^1^Division of Biology and Medicine, Department of Neuroscience, Brown University Providence, RI, United States; ^2^Division of Biology and Medicine, Department of Molecular Biology, Cell Biology and Biochemistry, Brown University Providence, RI, United States; ^3^Department of Neuroscience, Amgen Cambridge, MA, United States

**Keywords:** Genetic Inducible Fate Mapping (GIFM), *Gbx2*, cell fate decisions, cerebellum, Purkinje cells, granule neurons

## Abstract

The cerebellum (Cb) is an exquisite structure that controls elaborate motor behaviors and is essential for sensory-motor learning. During development, the Cb is derived from rhombomere 1 (r1). Within this embryonic compartment, precursors in r1 are patterned by signaling cues originating from the isthmus organizer (IsO) and subsequently undergo complex morphogenic movements to establish their final position in the mature Cb. The transcription factor *Gbx2* is expressed in the developing Cb and is intimately involved in organizing and patterning the Cb. Nevertheless, how precursors expressing *Gbx2* at specific embryonic time points contribute to distinct cell types in the adult Cb is unresolved. In this study, we used Genetic Inducible Fate Mapping (GIFM) to mark *Gbx2*-expressing precursors with fine temporal resolution and to subsequently track this lineage through embryogenesis. We then determined the terminal neuronal fate of the *Gbx2* lineage in the adult Cb. Our analysis demonstrates that the *Gbx2* lineage contributes to the Cb with marking over the course of five stages: Embryonic day 7.5 (E7.5) through E11.5. The *Gbx2* lineage gives rise to Purkinje cells, granule neurons, and deep cerebellar neurons across these marking stages. Notably, the contribution of the *Gbx2* lineage shifts as development proceeds with each marking stage producing a distinct profile of mature neurons in the adult Cb. These findings demonstrate the relationship between the temporal expression of *Gbx2* and the terminal cell fate of neurons in the Cb. Based on these results, *Gbx2* is critical to Cb development, not only for its well-defined role in positioning and maintaining the IsO, but also for guiding the development of Cb precursors and determining the identity of Cb neurons.

## Introduction

The cerebellum (Cb) controls motor behaviors, sensorimotor learning, gait, balance, coordination, proprioception, memory, reward, and cognition (reviewed in Zervas et al., 2005; Buckner, 2013; Wagner et al., 2017). The Cb forms over a prolonged developmental window, beginning during early embryogenesis and continuing postnatally (Altman and Bayer, 1997). This prolonged time course makes the Cb particularly vulnerable to developmental errors, which manifest in well characterized Cb disorders, including Dandy-Walker malformation, Joubert syndrome, Autism, and Tuberous Sclerosis (Ten Donkelaar and Lammens, 2009; Tsai et al., 2012; Reith et al., 2013; D'Mello and Stoodley, 2015). Thus, both basic scientific inquiry and emerging clinical interests encourage a deeper investigation into the cellular and molecular mechanisms underpinning Cb development.

During development, the Cb is derived from the anterior-dorsal region of rhombomere 1 (r1), which is patterned through a network of hierarchically organized and functionally interconnected transcription factors and secreted molecules (Zervas et al., 2005; Joyner and Zervas, 2006; Sillitoe and Joyner, 2007). *Gastrulation Brain Homeobox 2* (*Gbx2*) encodes a transcription factor that is integral for patterning the Cb (Li and Joyner, 2001). *Gbx2* is first expressed throughout the posterior extent of the embryo during gastrulation, but as development proceeds *Gbx2* expression becomes restricted to the spinal cord and r1 (Wassarman et al., 1997; Luu et al., 2011). *Gbx2* interacts with another homeobox transcription factor *Otx2*, which is expressed in the mesencephalon (mes). At their interface these two transcriptor factors exhibit mutual repression which initiates an intricate molecular pathway that establishes a signaling center at the mes/r1 boundary (Millet et al., 1999). This signaling center, known as the isthmus organizer (IsO), is delineated by the expression of *Fgf8* and is ultimately responsible for patterning both the presumptive midbrain and Cb (Liu and Joyner, 2001; Zervas et al., 2004; Sato and Joyner, 2009). Thus, *Gbx2* plays a critical role in Cb development, albeit indirectly, through its role in positioning and maintaining the IsO.

The functional requirement for *Gbx2* in Cb development was revealed by the striking phenotype of *Gbx2*^−/−^ mutant mice. In particular, *Gbx2*^−/−^ mutants have a complete loss of the Cb and die perinatally (Wassarman et al., 1997). A *Gbx2* conditional knockout (*Gbx2*-CKO) mouse line allowed for a more targeted deletion of *Gbx2*, which elucidated its temporal requirement in Cb development (Li et al., 2002). In contrast to *Gbx2*^−/−^ null mice, the conditional deletion of *Gbx2* allowed for the elimination of *Gbx2* specifically in r1 at temporally controlled and later stages in development (from E8.5 onward). Consequently, over half of *Gbx2*-CKO mutants survived into adulthood and produced Cb tissue. However, the remaining Cb did not develop normally nor establish conventional Cb structures. In particular, two distinct *Gbx2-CKO* phenotypes were observed: Severely affected *Gbx2*-CKO mutants were nearly devoid of a vermis, but had bi-lateral hemispheres. Less severely affected mutants had a distinct vermis, but displayed abnormally small vermal folia. Concomitant with these morphological changes, the genes *Otx2* and *Fgf8* were ectopically extended posteriorly into r1 (Li et al., 2002).

Thus, *Gbx2* is clearly required for the proper maintenance of the IsO and the subsequent patterning of the midbrain and anterior hindbrain. However, *Gbx2* may also shape the development of the Cb through cell autonomous mechanisms. Notably, the terminal cell fate of *Gbx2* expressing precursors and the distribution of their progeny has not been resolved in the Cb. Elucidating the *Gbx2* fate map would reveal the following information: 1. How the *Gbx2* lineage produces specific cell types in the Cb, 2. How the *Gbx2* lineage integrates into the mature structure of the Cb, and 3. Provide a more complete understanding of how *Gbx2* expression shapes Cb development. We addressed these gaps in the field using Genetic Inducible Fate Mapping (GIFM) to heritably mark and track cells with temporal control (Zervas et al., 2004; Joyner and Zervas, 2006; Ellisor et al., 2009). Based on *Gbx2*-CKO mice, we hypothesized that the *Gbx2* lineage would contribute to the Cb vermis more prominently than the lateral hemispheres. Moreover, we speculated that the down-regulation of *Gbx2* expression during development would result in the progressive restriction of the *Gbx2* lineage across development. In this report, we used *Gbx2-CreER* mice (Chen et al., 2009; Luu et al., 2011) to delineate temporally restricted fate maps of *Gbx2* derived neurons in the Cb. Specifically, we marked the *Gbx2* lineage at five distinct embryonic time points (E7.5-E11.5). Our fate mapping analysis did reveal medial biases, but we also uncovered additional differences in the spatial distribution and cell fate of *Gbx2*-derived neurons. Specifically, the *Gbx2* lineage produced numerous Cb cell types including neurons in the deep cerebellar nuclei (DCN), Purkinje cells, granule cells, and inhibitory interneurons. We also showed that the *Gbx2* lineage was not progressively restricted in its contribution to Cb cell types, suggesting that *Gbx2*-expressing precursors followed a competency model and generated distinct waves of cell type specific neurogenesis. Finally, the *Gbx2* lineage contribution was temporally and spatially complementary to the *Wnt1* lineage (Hagan and Zervas, 2012), which suggests that Cb precursors in the URL transition from a *Gbx2*+ molecular identity to a *Wnt1*+ identity in the URL. In this regard, the temporal regulation of *Gbx2* expression may have cell autonomous effects including the control of cell fate decisions in Cb precursors.

## Materials and methods

### Mice

*Gbx2*^*CreER*−*IRES*−*EGFP*/+^ mice (Chen et al., 2009) were generously provided by Dr. James Li (University of Connecticut Health Center). *Rosa26*^*tdTomato*^ reporter mice (*Ai14* allele, Madisen et al., 2010, referred to as *Rosa26*^*tdTomato*^ in this manuscript) were purchased from Jackson Laboratories (Stock No. 007908) and *Tau*^*mGFP*^ *(Tau-loxP-STOP-loxP-mGFP-IRES-NLS-LacZ-pA*, referred to as *mGFP* in this manuscript) reporter mice (Hippenmeyer et al., 2005) were generously provided by Dr. S. Arber (Jax lab, Stock No. 021162, www.informatics.jax.org/accession/MGI:3590682). Mice were housed and handled according to Brown University Institutional Animal Care and Use guidelines. Genotyping was done as previously described (Ellisor et al., 2009; Jackson Laboratories).

### GIFM and tissue preparation

Fate mapping experiments were conducted by crossing *Gbx2*^*CreER*−*IRES*−*EGFP*/+^*; Rosa26*^*tdTomato*^ or *Gbx2*^*CreER*−*IRES*−*EGFP*/+^*; mGFP* males with wildtype Swiss Webster females (Taconic). The morning (9:00 am) of the day a vaginal plug was detected was operationally defined as embryonic day (E) 0.5. Tamoxifen was administered at a dose of 4 mg to timed pregnant females by oral gavage (Brown et al., 2009; Ellisor and Zervas, 2010; Hagan and Zervas, 2012). Specifically, tamoxifen was administered at five distinct embryonic stages (E7.5, E8.5, E9.5, E10.5, or E11.5) and fate mapping tissue was collected at two different end points (E12.5 or postnatal day (P) 42. At E12.5, *Gbx2*^*CreER*−*IRES*−*EGFP*/+^*; Rosa26*^*tdTomato*^ fate mapped embryos were dissected in PBS over ice, fixed in 4% paraformaldehyde (PFA) overnight at 4°C, cryoprotected, and embedded in OCT. Embryos were sectioned sagittally (12 μm thickness) with a Leica cryostat and stored at −20°C. At P42, *Gbx2*^*CreER*−*IRES*−*EGFP*/+^*; mGFP* fate mapped mice were deeply anesthetized with Nembutal and intracardially perfused with 4% PFA. Craniotomies were performed and fate mapped brains were placed in 4% PFA and sectioned sagittally (40 μm) using a Leica vibratome. Three fate mapped brains across two litters were processed for each marking stage and analysis stage.

### Immunocytochemistry (ICC)

Tissue sections were analyzed by ICC as previously described (Ellisor et al., 2009; Hagan and Zervas, 2012). The following primary antibodies were used: rabbit anti-DsRed (1:500 Clontech, Cat # 632496), goat anti-ß-galactosidase (ß-gal, 1:500, Biogenesis, Catalog #4600-1409), chick anti-ß-galactosidase (1:500, Abcam, Catalog #ab9361-250), rabbit anti-GFP (1:600, Molecular Probes; Carlsbad, CA; Catalog #A-11122), rabbit anti-calbindin (1:1,000, Swant, Catalog #CB3a), goat anti-calretinin (1:5,000, Chemicon; Billerica, MA; Catalog #AB1550), mouse anti-parvalbumin (1:1,000, Sigma, Catalog #P3088-0.2 ML), and rabbit anti-Pax2 (1:50, Invitrogen, Catalog #71-6000). Secondary antibodies [Alexa 555 (Invitrogen: Cat #A-31572, donkey anti-rabbit IgG; Catalog #A-31570 donkey anti-mouse IgG; Catalog #A21432, donkey anti-goat IgG), Dylight 549 (Jackson ImmunoResearch Laboratories; Catalog #703-505-155, donkey anti-chicken), Coumarin AMCA (Jackson ImmunoResearch Laboratories: Catalog #703-155-155, donkey anti-chicken), and Alexa 488 (Invitrogen: Catalog #A21208, donkey anti-rabbit IgG; Catalog #A-21208, donkey anti-rat IgG; Catalog #A21202, donkey anti-mouse IgG)] were prepared at 1:500.

### Microscopy

Data were collected with a Leica DM6000 B epifluorescent microscope using Volocity 5.1 imaging software (Improvision). Low magnification images were captured with 2.5× and 5× objectives and high magnification images were obtained using a motorized stage with 10×, 20×, or 40× objectives. True magnifications are indicated in figures by scale bars. All images were pseudo colored live as part of the acquisition palettes. Imaging data sets were exported to Adobe Photoshop CS6 where montages of representative data were generated. Illustrations were generated using Adobe Illustrator CS6.

## Results

### Timing of *Gbx2* controls the spatial distribution of the *Gbx2* lineage in the developing Cb

**Figure 1:**
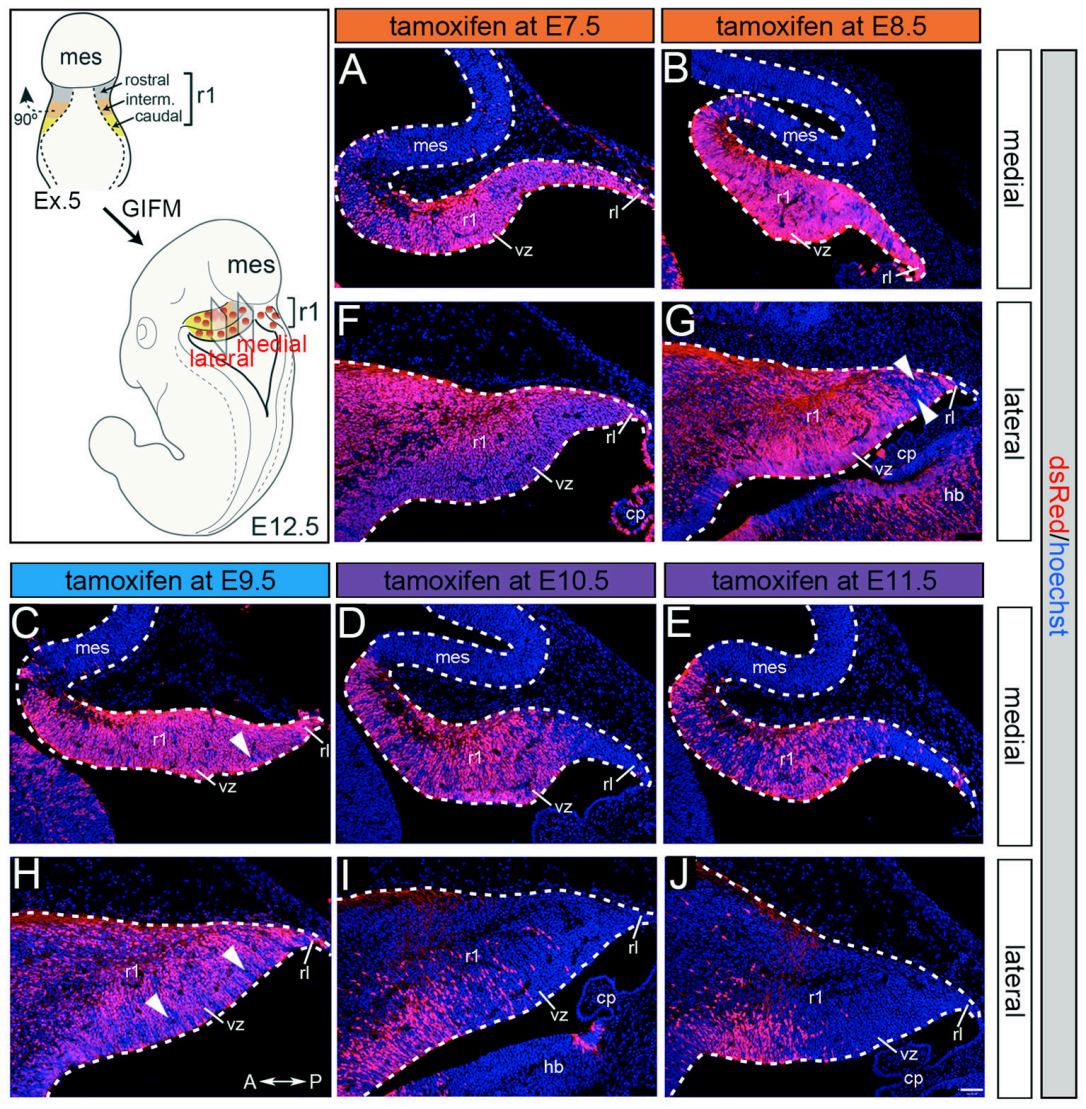
*Gbx2* expressing precursors differentially populate the Cb primordia. ICC with an antibody recognizing tdTomato (DsRed, red is produced from recombination of the *Rosa26*^*tdTomato*^ allele) on E12.5 fate mapped sagittal sections. **(A,F)** The *Gbx2* lineage marked at E7.5 gave rise to a majority of cells in the ventricular zone (vz), rhombic lip (rl), and core of rhombomere 1 (r1) in medial and lateral r1. Notably, a small number of *Gbx2*-derived cells was observed in the mesencephalon (mes). **(B,G)** The *Gbx2* lineage marked at E8.5 also contributed to cells throughout the vz and rl in the Cb primordia. **(C,H)** The *Gbx2* lineage marked at E9.5 produced the largest contribution of cells in medial r1. However, cohorts of cells with clonal-like labeling was apparent in the vz of lateral r1. **(D,I)** The *Gbx2* lineage marked at E10.5 preferentially populated the anterior aspect of medial r1, but was nearly absent in the rl. In contrast, lateral r1 was devoid of labeling in the core differentiated zone and the rl. There was also diminished labeling in the posterior vz. **(E,J)** The *Gbx2* lineage marked at E11.5 was diminished in its contribution throughout medial r1 and marked cells appeared clonal-like in nature. Only a small population of marked cells was observed in the anterior-lateral vz. White arrowheads indicate unmarked cohorts of cells. Scale bar: 61 μm. See Supplemental Figure 1 for fate mapping schematic summarizing alleles and approach.

**Figure 2:**
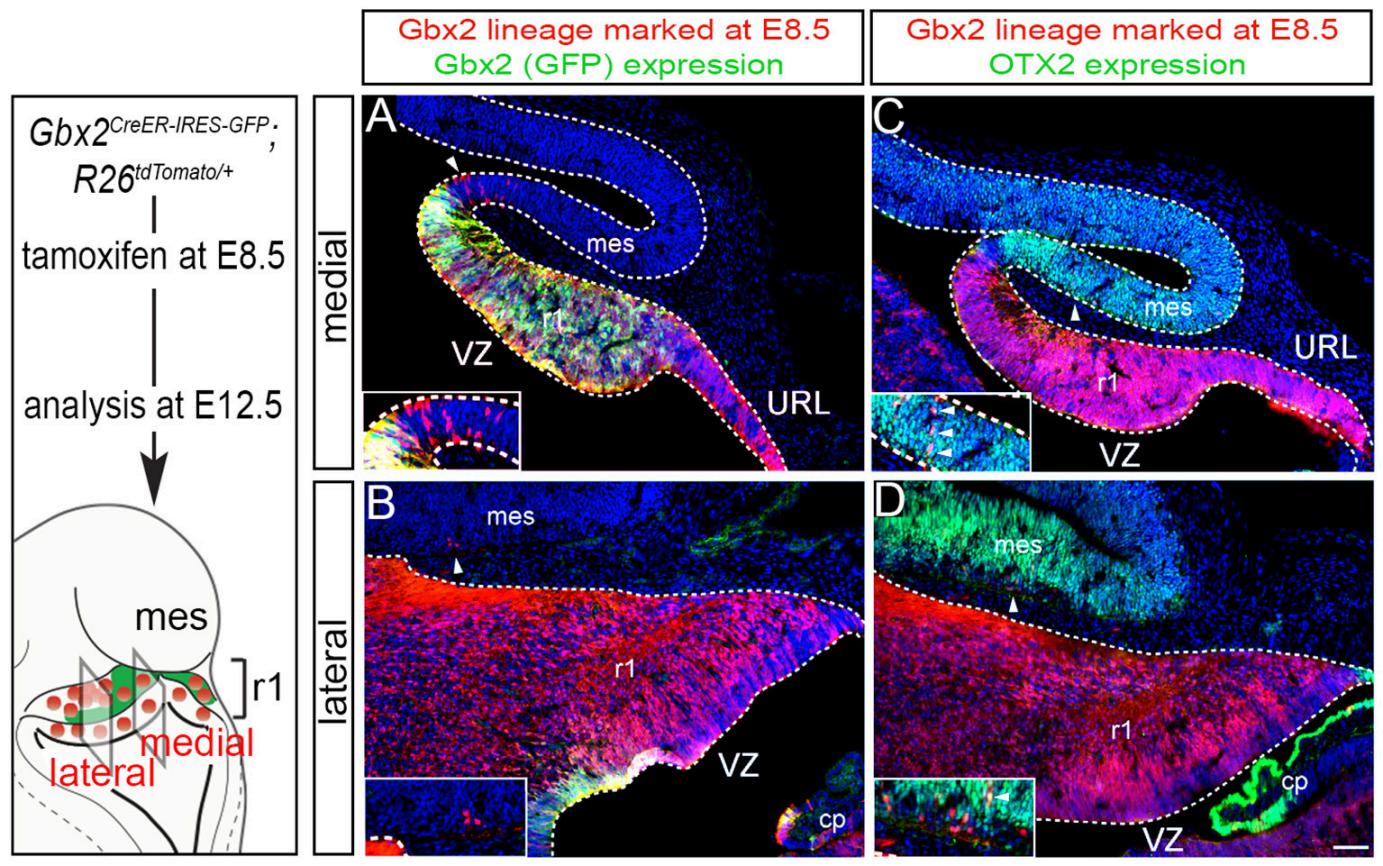
Dynamic *Gbx2* expression within the *Gbx2* lineage in the Cb primordia. **(A,B)** Sections obtained from E12.5 *Gbx2*^*CreER*−*IRES*−*EGFP*/+^; *Rosa26*^*tdTomato*^ embryos. ICC was performed with an antibody recognizing tdTomato, which reveals the *Gbx2* lineage marked at E8.5 (DsRed, red) concomitant with GFP labeling to reveal current *Gbx2* expression (GFP, green). Note that *Gbx2* expression was nearly absent in lateral r1 while *Gbx2* expression was still present in medial sections with the exception of the posterior domain. **(C,D)** Sections adjacent to those shown in **(A,B)** were immunolabeled for tdTomato (DsRed, red) to identify the *Gbx2* lineage and for OTX2 (green), which is a marker of the mes. Clones of the *Gbx2* lineage were occasionally observed in the OTX2+ mes (arrowheads, inset).

Although *Gbx2* expression throughout development has previously been described, the allocation of *Gbx2*-derived precursors within the Cb primordia has not been determined. Therefore, we used GIFM (Joyner and Zervas, 2006; Dymecki and Kim, 2007) concomitantly with *Gbx2*^*CreER*−*IRES*−*EGFP*/+^; *Rosa26*^*tdTomato*^ mice to permanently and heritably mark *Gbx2*-expressing precursors. Marked cells express tdTomato (red fluorescence is produced from recombination of the *Rosa26*^*tdTomato*^ allele). Specifically, we administered tamoxifen to *Gbx2*^*CreER*−*IRES*−*EGFP*/+^; *Rosa26*^*tdTomato*^ embryos at five distinct developmental stages (E7.5, E8.5, E9.5, E10.5, and E11.5) and then analyzed embryos at E12.5 (Figure 1). With early marking (tamoxifen at E7.5 or E8.5), the *Gbx2* lineage gave rise to a majority of cells in r1 at E12.5 (Figures 1A,F,B,G). However, small populations of unlabeled cells were observed in the ventricular zone (VZ) of the lateral-posterior region of r1 with marking at E8.5 (Figure 1G, arrowheads). The *Gbx2* lineage marked at E9.5 contributed to cells throughout medial and intermediate sagittal planes of r1. However, increased domains of unlabeled cells were distributed along the anterior-posterior (A-P) axis in lateral r1 and were more extensive compared to marking a day earlier (Figure 1H, arrowheads). Finally, at later marking stages (E10.5-E11.5) we observe two notable alterations: 1. The *Gbx2* lineage preferentially populated anterior r1 and showed a sharp decrease in the contribution to posterior r1; this was observed across the entire medial-lateral extent of the embryonic Cb (Figures 1D,I,E,J); 2. The *Gbx2* lineage became even more sparse in the lateral VZ at these later stages (Figures 1I,J). Notably, only sparse *Gbx2*-derived cells were observed in the upper rhombic lip and the posterior ventricular zone (Figures 1D,I,E,J). In lateral r1, *Gbx2*-derived cells were further segregated ventrally (Figure 1J). Thus, *Gbx2* was extinguished in r1 in a posterior-lateral to anterior-medial direction.

We next took advantage of *Gbx2*^*CreER*−*IRES*−*EGFP*/+^; *Rosa26*^*tdTomato*^ mice to mark the *Gbx2* lineage at E8.5 and assess the lineage concomitantly with *Gbx2* expression (EGFP) at E12.5 (Figure 2). This allowed us to compare how *Gbx2* expression was regulated within the *Gbx2* lineage as the Cb architecture was being established. In both medial and lateral sections at E12.5, the *Gbx2* lineage that had been marked by tamoxifen administration at E8.5 was distributed throughout r1 including the URL (Figures 2A,B, red labeling), which is consistent with data shown in Figure 1. In the URL of posterior-medial r1 the *Gbx2* lineage was present, but no longer continued to express *Gbx2* (Figure 2A, URL). The dorsal mes and r1 are separated by the IsO, which expresses *Fgf8* (Zervas et al., 2004). This translates into the IsO being framed by the expression of OTX2 (marker of the mes) and GBX2 (marker of r1), (Figures 2A,B). Notably, a small number of cells derived from the *Gbx2* lineage was observed in the IsO (Figure 2A, white arrowhead and inset) and had ceased to express *Gbx2* (EGFP, green). *Gbx2*-derived cells with a clonal arrangement were also occasionally observed in the medial mes at E12.5 (Figure 2C, white arrowheads, inset).

In lateral sections, *Gbx2* expression had extinguished between E8.5 and E12.5 throughout r1 with the exception of a small domain in the VZ (Figure 2B). Although the *Gbx2* lineage was largely confined to r1, small clones could be observed in the mes (Figure 2B, white arrowhead, inset). *Gbx2*-derived cells could also be seen in the lateral OTX2+ mes, yet most of the *Gbx2* lineage in this territory did not yet express OTX2 (Figure 2D, white arrowhead, inset). However, a rare example of cells derived from the *Gbx2* lineage (red) was observed in the mes and was coincident with OTX2 expression (Figure 2D, yellow cell, inset) suggesting that the small population derived from the *Gbx2* lineage that entered the mes between E8.5-E12.5 were in the processing of dynamically switching their molecular identity to that of a mesencephalic cell.

### The *Gbx2* lineage differentially contributes to the granule cell layer

As development proceeds, Cb progenitors migrate, differentiate, and acquire their mature molecular identity. Therefore, we followed *Gbx2*-expressing progenitors across development to determine their terminal cell fate in the adult Cb. We used GIFM, taking advantage of *Gbx2*^*CreER*−*IRES*−*EGFP*/+^ mice and the conditional *mGFP* reporter line, which upon recombination produces both nuclear beta-galactosidase and EGFP (Supplemental Figure 1), to permanently and heritably mark *Gbx2*-expressing precursors at distinct time points during embryogenesis. Subsequently, we analyzed the distribution of the *Gbx2* lineage in the mature Cb. The *mGFP* reporter line allowed us to identify the *Gbx2* lineage via their expression of nuclear LacZ and membrane bound GFP. Through this analysis, we showed that the *Gbx2* lineage gave rise to a variety of neuronal cell types in the mature Cb, with a striking contribution to granule cells.

**Figure 3:**
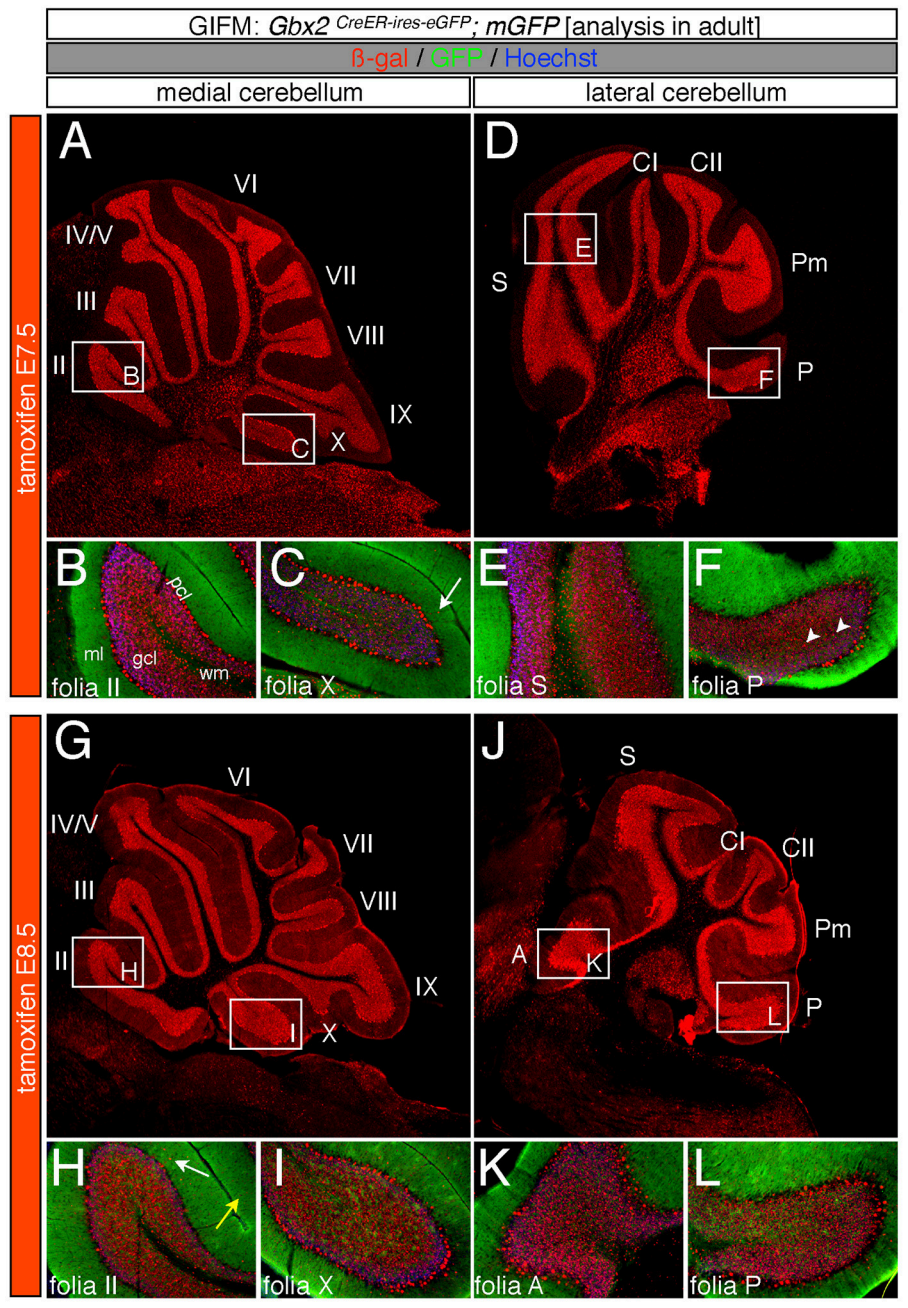
Spatial and temporal contribution of the early marked *Gbx2* lineage to the mature Cb. ICC on adult sections with antibodies that recognize ß-gal (red) or GFP (green) revealed that early *Gbx2* expressing progenitors give rise to cells throughout the Cb cortex. **(A–F)** *Gbx2* expressing progenitors marked at E7.5 substantially contributed to granule cells. Medially, *Gbx2*-derived granule cells preferentially populated the most anterior folia **(A–C)** while laterally, the *Gbx2* lineage was more evenly distributed across the Cb **(D–F)**. The *Gbx2* lineage also gave rise to Purkinje cells at this marking stage, but the contribution was biased toward the medial posterior Cb **(B,C,E,F)**. **(G–L)** *Gbx2* expressing progenitors marked at E8.5 were evenly distributed throughout the Cb and constituted the peak contribution to granule cells. Similar to marking a day earlier, *Gbx2* expressing progenitors marked at E8.5 also produced Purkinje cells, particularly in the medial posterior Cb **(H,I,K,L)**. White arrows demarcate Gbx2-derived nuclei in the molecular layer and arrowheads indicate “intermediate cells” in the granule cell layer and white matter. Scale bars: 180 μm **(A,D,G,J)**, 90 μm **(B,C,E,F,H,I,K,L)**. See Supplemental Figure 2 for fate mapping summary of *Gbx2* lineage contribution to granule cells when marked early.

Specifically, when tamoxifen was administered at E7.5, the *Gbx2* lineage contributed to granule cells both in the medial (vermis) and lateral hemispheres (Figures 3A–F). Medially, *Gbx2*-derived granule cells were most prominent in the central zone (lobules VI and VII) and least pronounced in the nodular zone (lobules X and ventral IX) (Figure 3A). This spatial bias was evident by comparing the anterior folia (folia II) and the posterior folia (folia X) of the vermis at higher magnification (Figures 3B,C). Laterally, *Gbx2*-derived granule cells were more evenly distributed across all folia and contributed heavily to granule cells in the paramedian lobule (Pm) and contributed to numerous granule cells in the lobulus simplex (S), crus I (CI), crus II (CII), and copula pyramidis (P) (Figures 3D–F). Similarly, progenitors expressing *Gbx2* at E8.5 contributed to granule cells in both the vermis and hemispheres (Figures 3G–L). Notably, the *Gbx2* lineage marked at E8.5 constituted the peak contribution to granule cells across the medial-lateral axis. In the vermis, the anterior bias that was observed with marking a day earlier (at E7.5) was no longer present and robust granule cell contribution was observed throughout the Cb (Figures 3G–I). Laterally, the *Gbx2* lineage contributed strongly to Pm, but also populated the other lobules (Figures 3J–L).

**Figure 4:**
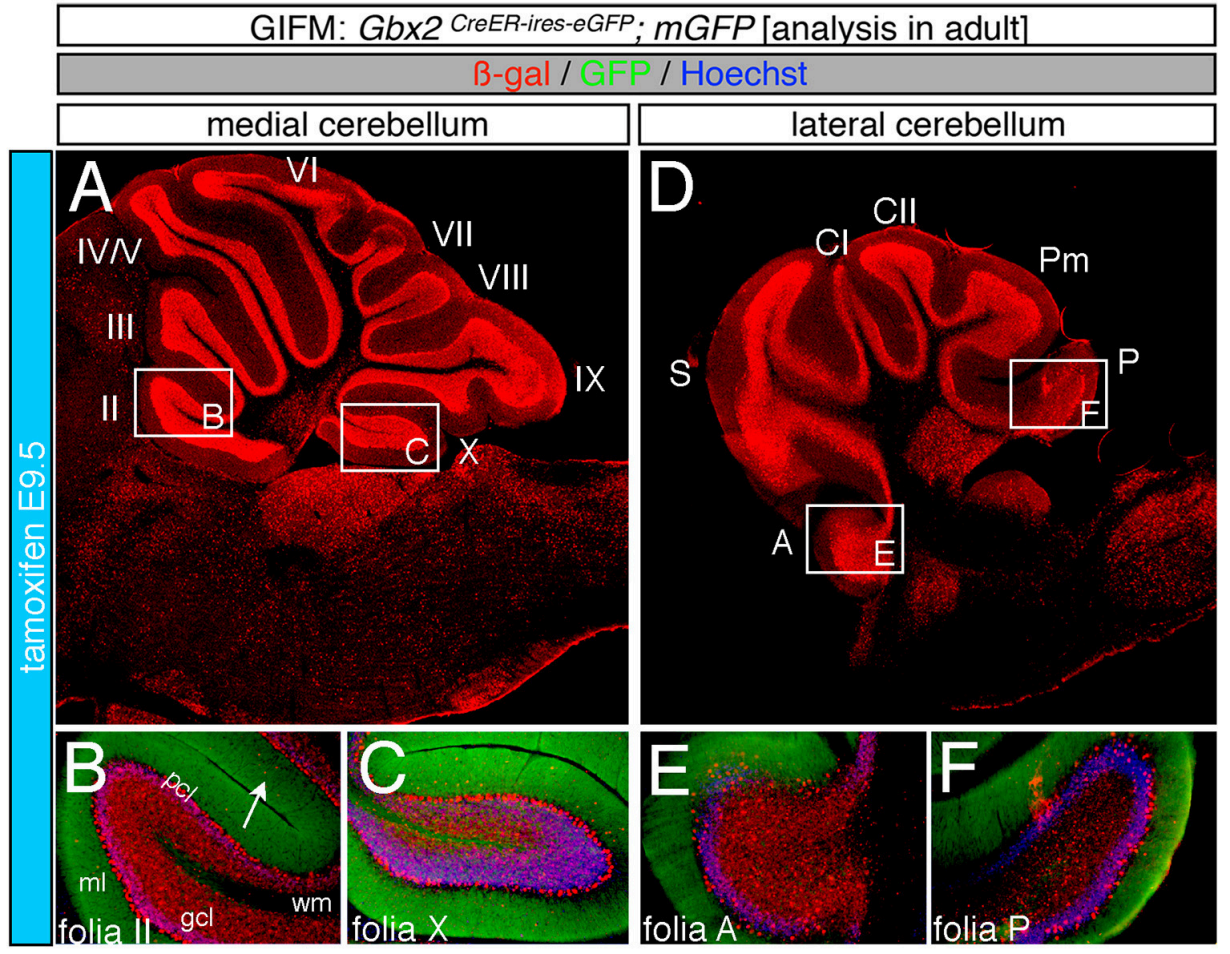
Progenitors expressing *Gbx2* at E9.5 delineates the peak contribution to granule cells in the adult Cb. ICC on adult sections with antibodies that label ß-gal (red) and GFP (green). **(A–F)** *Gbx2* expressing progenitors marked at E9.5 produced fewer granule cells than marking at earlier time points. These Gbx2-derived granule cells were predominantly localized to the anterior Cb, with a substantial bias toward the anterior hemispheres **(E)**. This intermediate marking stage also generated the peak *Gbx2* lineage contribution to Purkinje cells. *Gbx2*-derived Purkinje cells populated every folia, but the greatest contribution was observed in the posterior vermis. White arrow demarcates *Gbx2*-derived nuclei in the molecular layer. Scale bar: 180 μm **(A,D)**, 90 μm **(B,C,E,F)**. See Supplemental Figure 2 for fate mapping summary of *Gbx2* lineage contribution to granule cells when marked at an intermediate stage.

**Figure 5:**
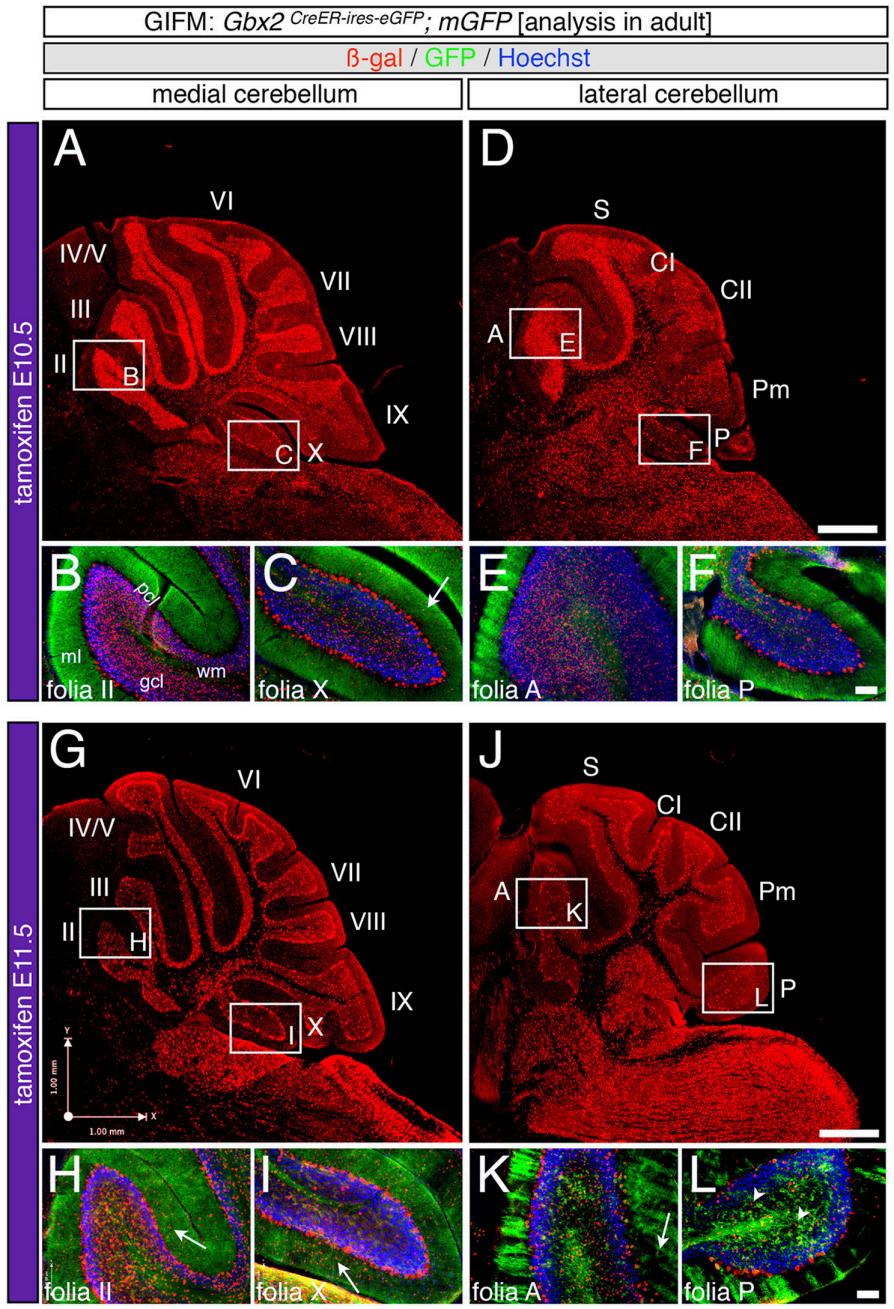
Precursors expressing *Gbx2* late give rise to few granule cells and Purkinje neurons in the adult Cb. ICC on adult sections with antibodies that label ß-gal (red) and GFP (green). **(A–F)** *Gbx2* expressing progenitors continued to produce granule cells with marking at E10.5. However, a significant decrease in granule cell labeling in the posterior Cb created an anterior bias at this marking stage **(B,C,E,F)**. *Gbx2*-derived Purkinje cells were also observed with marking at E10.5 and preferentially populated the posterior vermis **(C)**. **(G–L)** The *Gbx2* lineage persisted in giving rise to granule cells with marking at E11.5. However, this late contribution was significantly reduced compared to earlier marking stages and continued to be biased toward the anterior Cb **(H,I,K,L)**. *Gbx2*-derived Purkinje cells were still observed by marking at E11.5 and continued to be predominately located in the posterior vermis **(I)**. White arrows demarcate *Gbx2*-derived nuclei in the molecular layer and arrowheads indicate “intermediate cells” in the granule cell layer and white matter. Scale bars: 180 μm **(A,D,G,J)**, 90 μm **(B,C,E,F,H,I,K,L)**. See Supplemental Figure 2 for fate mapping summary of *Gbx2* lineage contribution to granule cells when marked late.

Marking at mid-embryonic development (E9.5) revealed that *Gbx2*-expressing progenitors persisted in giving rise to granule cells throughout the Cb (Figure 4). With marking at E9.5, the *Gbx2* lineage populated the anterior vermis with a subtle decrease in *Gbx2*-derived granule cells in the most posterior folia of the vermis (compare Figures 3H,I to Figures 4B,C). In contrast, there was a noticeable decrease to the posterior folia in the bi-lateral hemispheres compared to marking a day earlier (compare Figures 3J–L to Figures 4D–F). We subsequently labeled and followed the *Gbx2* lineage at later stages and showed that the *Gbx2* lineage persisted in contributing to Cb granule cells (Figure 5). Marking at E10.5 showed that the *Gbx2* lineage preferentially populated the anterior vermis while far fewer *Gbx2*-derived granule cells were observed posteriorly (Figures 5A–C). In particular, the most prominent contribution to granule cells with marking at E10.5 was seen in the anterior zone (lobules I-V). Notably, granule cell marking was progressively diminished in the central zone (lobules VI and VII) and the posterior zone (lobules VIII and dorsal IX). The most significantly decreased contribution was to the nodular zone (dorsal IX and X) (Figure 5C). The anterior bias was also observed in the lateral hemispheres (Figures 5D–F). In general, anterior lobules (A, S, and CI) contained more fate mapped granule cells than posterior lobules (CII, Pm, and P) with marking at E10.5. The *Gbx2* lineage gave rise to the greatest number of granule cells in anterior folia, such as the lobulus simplex (S) and the fewest in copula pyramidis (P) with marking at E10.5 (Figures 5E,F). By comparing the medial vermis and lateral hemispheres, there was a clear medial bias toward *Gbx2*-derived granule cells (Figures 5A,D). This bias was most noticeable in the posterior Cb, with the posterior hemispheres exhibiting a significant decrease in *Gbx2*-derived granule cells compared to the posterior vermis (Figures 5C,F). Marking at E10.5 also revealed that the *Gbx2* lineage was significantly shifted compared to earlier marking stages with fewer labeled granule cell projections in the molecular layer which consequently revealed a slightly banded marking pattern in the lateral hemispheres (Figures 5E,F). The molecular layer was comprised of a richly dense, and uniform plexus of GFP-positive projections due to the large number of granule cell axons with early marking (see Figures 3B,C,E,F,H,I,K,L). Finally, marking at E11.5 revealed that the *Gbx2* lineage gave rise to fewer granule cells vs. any other other earlier marking stage (Figures 5G–L). In the vermis, the reduced amount of *Gbx2*-derived granule cells was observed in the anterior lobe (Figures 5G,H) while the posterior vermis was nearly devoid of *Gbx2*-derived granule cells (Figures 5G,I). Marking the *Gbx2* lineage at E11.5 resulted in only sparse labeling of granule cells and parallel fibers in the molecular layer of Cb hemispheres (Figures 5J–L). Consequently, Purkinje cell dendrites were observed as a discernible striped projection pattern in the molecular layer (Figures 5K,L). Marking at this later stage also revealed *Gbx2*-derived neurons in the molecular layer of the vermis (Figures 5H,I, arrows). A summary of *Gbx2* lineage contribution to granule cells in the Cb appears in Supplemental Figure 2.

### The *Gbx2* lineage produces purkinje cells over a prolonged time period

**Figure 6:**
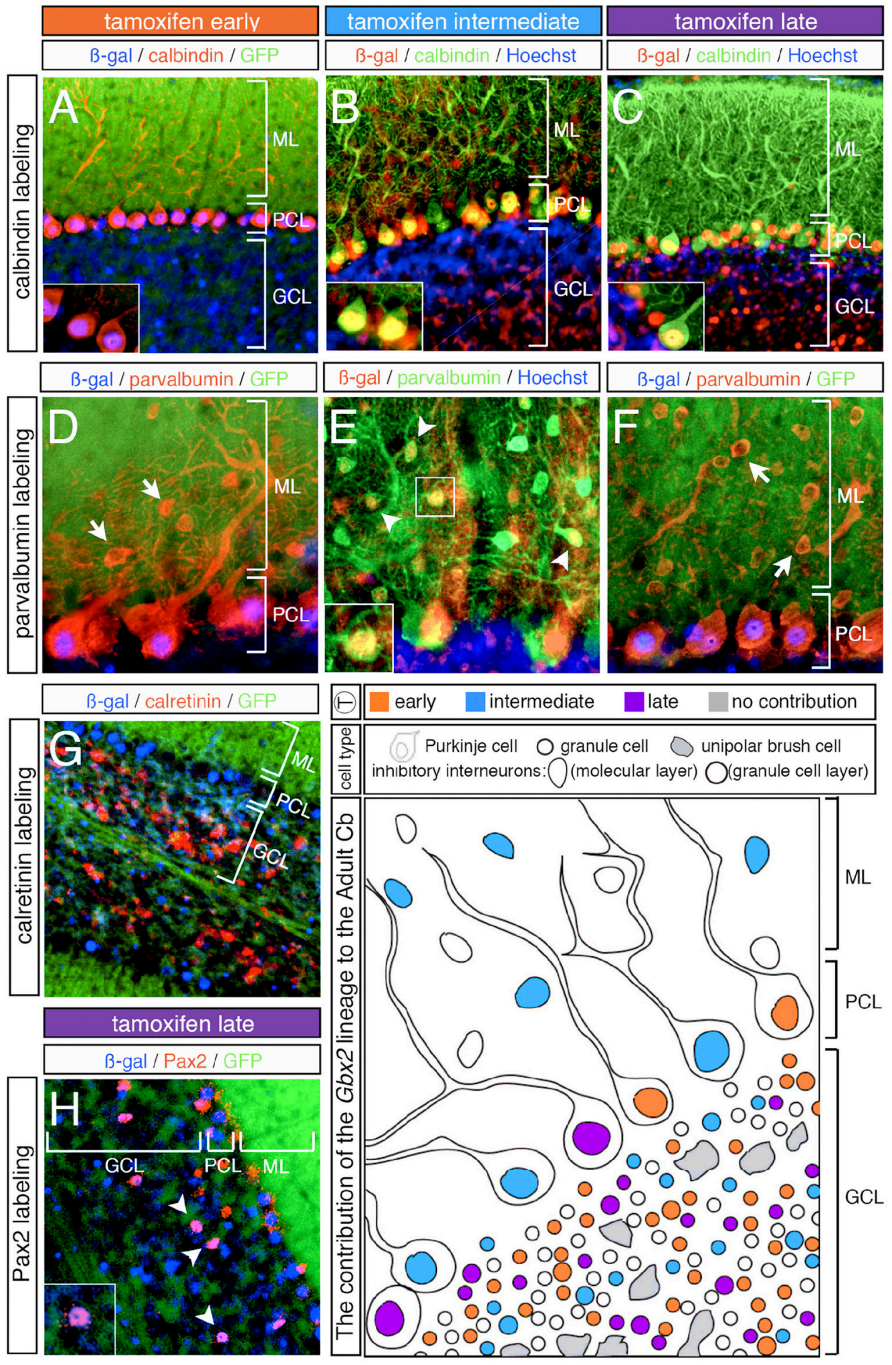
Overview of Purkinje cell marker analysis in the *Gbx2* lineage. ICC on adult sagittal sections with antibodies against ß-gal or GFP and cell type specific biomarkers. **(A–C)** *Gbx2* expressing progenitors contributed to calbindin+ Purkinje cells at all stages of marking. **(D–F)** The *Gbx2* lineage gave rise to parvalbumin+ Purkinje cells at all stages of marking, but only produced parvalbumin+ inhibitory interneurons with marking at E9.5. **(G)** *Gbx2* expressing progenitors failed to contribute to calretinin+ unipolar brush cells all stages of marking. **(H)** The *Gbx2* lineage gave rise to a few Pax2+ inhibitory interneurons at all stages of marking. Schematic summarizing the contribution of the *Gbx2* lineage to granule cells, Purkinje cells, inhibitory interneurons, and unipolar brush cells at early (E7.5-E8.5, orange), intermediate (E9.5, blue) and late (E10.5-E11.5, purple) marking stages. Abbreviations: ML, molecular layer; PCL, Purkinje cell layer; GCL, granule cell layer. Scale bars: 90 μm **(A–C,G)**, 46 μm **(D–F,H)**. See Supplemental Figure 3 for fate mapping summary of *Gbx2* lineage contribution to Purkinje cells at early, intermediate, and late marking stages.

In addition to neurons in the granular cell layer, large *Gbx2*-derived neurons were observed in the Purkinje cell layer at every marking stage (Figures 3–5). With early marking (tamoxifen at E7.5 and E8.5), the *Gbx2* lineage contributed to Purkinje cells throughout the vermis and hemispheres, with a bias toward the posterior vermis (Figure 3). There was also a posterior bias of *Gbx2*-derived Purkinje cells in the vermis observed with marking at E9.5 (Figures 4A–C). We verified that these *Gbx2*-derived neurons were indeed Purkinje cells by co-localizing ß-gal with two different Purkinje cell biomarkers, calbindin and parvalbumin (Figures 6A–F). Calbindin is a calcium binding protein expressed specifically in Purkinje cells in the Cb while parvalbumin is also a calcium binding protein, but is expressed in both Purkinje cells and inhibitory interneurons of the molecular layer (Bastianelli, 2003). Our biomarker analysis confirmed that nearly all calbindin+ (Figures 6A–C) and PV+ (Figures 6D–F) Purkinje cells were ßgal+. These results indicate that the *Gbx2* lineage marked at E9.5 represents the peak contribution to Purkinje cells (Figures 6B,E). Finally, with later marking (E10.5 and E11.5), the *Gbx2* lineage continued to give rise to Purkinje cells. However, there was a prominent decrease in *Gbx2*-derived Purkinje cells in the lateral-posterior Cb (Figure 5). This observation was confirmed with calbindin and parvalbumin marker analysis (data not shown). A summary of *Gbx2* lineage contribution to Purkinje cells in the Cb appears in Supplemental Figure 3.

### The *Gbx2* lineage has a minimal contribution to cerebellar interneurons

In our fate mapping experiments, we observed *Gbx2*-derived neurons in both the granular and molecular layers that could not be identified based on cellular morphology alone. Therefore, we again used cell-type specific biomarkers in conjunction with ß-gal to determine the molecular identity of these cells. First, we used parvalbumin to discern GABAergic interneurons positioned in the molecular layer (ML). With both early (E7.5 and E8.5) and late (E10.5 and E11.5) marking stages, the *Gbx2* lineage failed to give rise to any parvalbumin+ cells in the ML (Figures 6D,F, arrows). It is noteworthy that *Gbx2*-derived inhibitory interneurons (parvalbumin+) were observed in the molecular layer of all folia, but only with marking at E9.5 (Figure 6E, arrowheads, inset). Interestingly, this intermediate marking stage corresponded to the peak contribution of the *Gbx2* lineage to another GABAergic cell type, Purkinje cells, which were positioned in the Purkinje cell layer (PCL) (Figures 6D–F).

We observed Gbx2-derived neurons in the granule cell layer (GCL) that were larger than granules cells yet smaller than Purkinje cells. Based on their size and location in the granule cell layer, we assessed whether these *Gbx2*-derived “intermediate” cells expressed calretinin, a calcium binding protein expressed in a subpopulation of unipolar brush cells (Englund et al., 2006). In particular, unipolar brush cells are glutamatergic interneurons located in the nodular zone of the vermis (Bastianelli, 2003). However, the *Gbx2* lineage failed to give rise to calretinin+ cells at any marking stage (Figure 6G; data not shown). In contrast, GABAergic interneurons positioned in the GCL are identified by the expression of *Pax2*, a paired box transcription factor (Maricich and Herrup, 1999). At all marking stages, the *Gbx2* lineage gave rise to relatively few Pax2+ cells medially and laterally (Figure 6H, white arrowheads).

**Figure 7:**
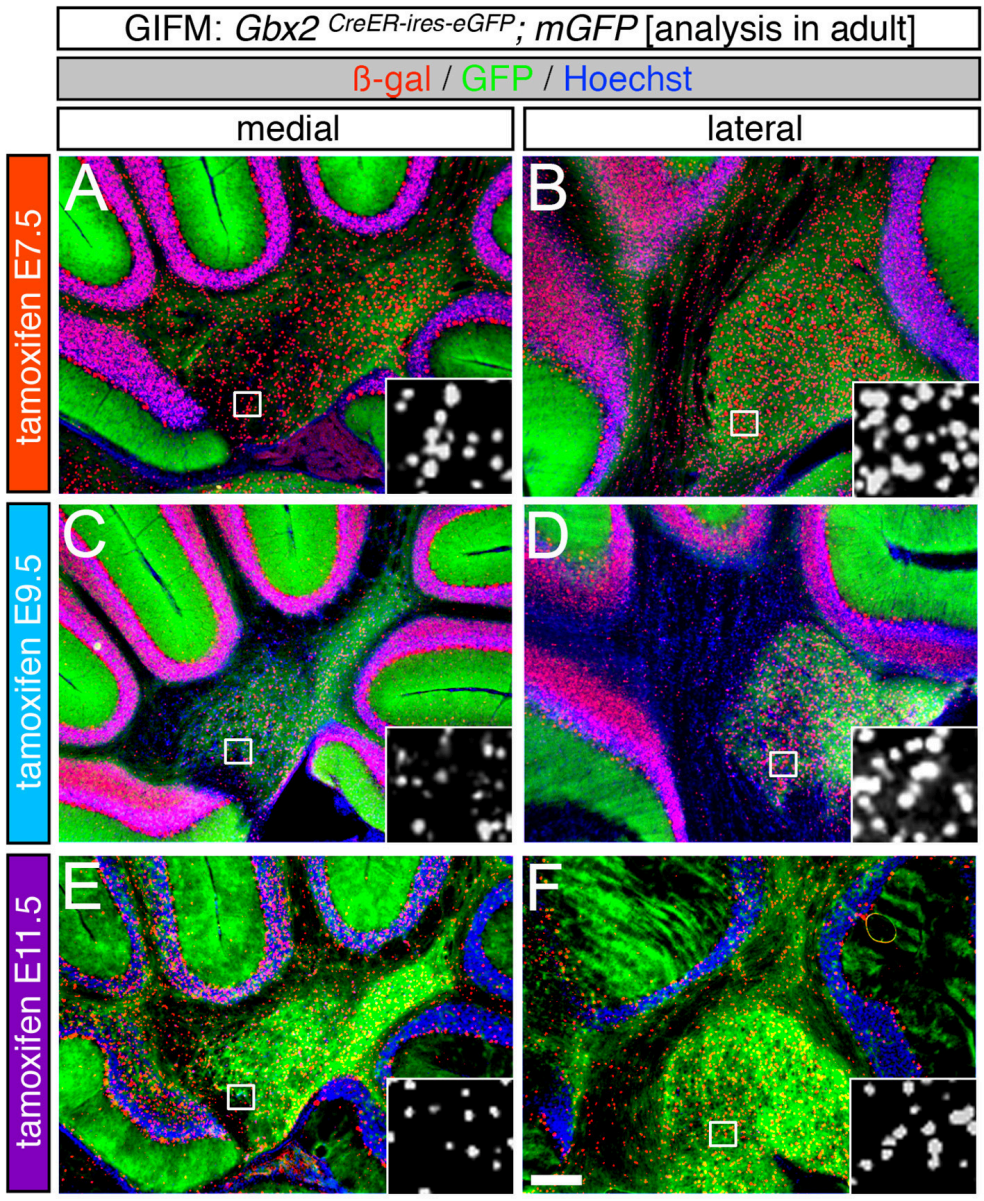
The *Gbx2* lineage gives rise to DCN neurons at all five marking stages. ICC on adult sections with antibodies that recognize ß-gal (red) or GFP (green) revealed that *Gbx2* expressing precursors contributed to all three DCN. **(A–F)** Gbx2-derived cells were found in both the medial **(A,C,E)** and lateral **(B,D,F)** DCN at every marking stage. Early *Gbx2* expressing precursors (E7.5, **A,B**) contributed to the DCN more substantially than all subsequent fate mapping stages **(C–F)**. Scale bar: 180 μm.

Finally, the *Gbx2* lineage gave rise to neurons in the DCN with marking at each embryonic stage in our analysis (Figure 7). The *Gbx2* lineage had its highest contribution to DCN neurons with marking at E7.5 (Figures 7A,B). With this early marking stage, *Gbx2*-derived neurons were found evenly distributed in all three DCN. At intermediate marking stages (tamoxifen at E9.5), there was a sharp drop off of medially marked neurons vs. lateral DCN neurons (Figures 7C,D). However, with late marking (tamoxifen at E11.5), there were notably fewer *Gbx2*-derived neurons located in lateral DCN than intermediate or medial nuclei (the interpositus and fastigial nuclei, respectively) (Figures 7E,F).

## Discussion

The homeobox transcription factor, *Gbx2*, is transiently expressed during embryogenesis and functions to position and maintain the IsO, which is located at the junction of the mes and r1. In addition, *Gbx2* plays an integral role in patterning the presumptive Cb. However, the cell autonomous lineage decisions of *Gbx2* expressing precursors has not been investigated in the Cb. Additionally, analysis of *Gbx2* expression in r1 does not uncover the relationship between the timing of *Gbx2* expression and terminal cell fate decisions of Cb precursors nor does it reveal the contribution of *Gbx2* expressing precursors to the mature Cb. With GIFM, we permanently and heritably marked *Gbx2* expressing precursors at specific embryonic stages and tracked these precursors and their progeny across development. Our GIFM analysis reveals that the *Gbx2* lineage is not progressively restricted in its contribution to the Cb, but rather gives rise to molecularly and spatially distinct subpopulations at different developmental stages (summarized in Figure 6). These findings raise the possibility that *Gbx2* expression guides Cb development from within r1 precursors and that the previously reported *Gbx2*^−/−^ phenotype results, not only from perturbations in the IsO, but also from a loss of cell autonomous *Gbx2* function (i.e., cell fate decisions).

At E8.5 *Gbx2* is expressed throughout the entire extent of r1, but by E9.5 the *Gbx2* expression domain becomes more restricted spatially (Liu and Joyner, 2001). Based on this expression pattern, we hypothesized that the *Gbx2* lineage would contribute to all Cb neurons with early marking and that this contribution would diminish with marking at intermediate and later stages. However, we discovered that the *Gbx2* lineage gives rise to several Cb cell types with distinct peaks of contribution. For example, the greatest contribution to the DCN occurs at E7.5 (Figure 7). In contrast, the peak contribution to granule cells occurs at E8.5 (Figure 3) and the peak contribution to Purkinje cells transpires at E9.5 (Figures 4, 6). These observations disprove that a lineage restriction model applies to *Gbx2* expressing progenitors and indicates that *Gbx2* is induced *de novo* in Cb progenitors at distinct temporal epochs.

Neurogenesis within the Cb is segregated into two distinct germinal zones during development: 1. The ventricular zone (VZ), which produces all inhibitory neurons of the Cb; 2. The upper rhombic lip (URL), which generates all excitatory Cb neurons (Hoshino et al., 2005; Machold and Fishell, 2005; Wang et al., 2005; Leto et al., 2006). Within each germinal zone, neurogenesis is also temporally compartmentalized resulting in different Cb cell types emerging at distinct embryonic stages. Our GIFM analysis of adult mice demonstrates that the *Gbx2* lineage yields substantial production of DCN neurons, granule cells, and Purkinje cells. In contrast, the *Gbx2* lineage makes only a minor contribution to inhibitory interneurons of the granular and molecular layers and does not give rise to unipolar brush cells. These results indicate that *Gbx2* expressing precursors contribute to the earliest born cell types produced in the Cb germinal zones. It also appears that Cb cell types generated later in embryogenesis (post-E11.5) do not have a history of *Gbx2* expression. Interestingly, *Gbx2*-derived granule cells have a biased contribution to the anterior-medial Cb where the first born granule cells are located (Altman and Bayer, 1997). Similarly, *Gbx2*-derived Purkinje cells are predominately found in the posterior Cb where the earliest born Purkinje cells are positioned (Altman and Bayer, 1997). These findings suggest that progenitors within the VZ and URL lip are temporally and molecularly partitioned into two distinct cohorts (*Gbx2*+ and *Gbx2*-). Thus, *Gbx2* expression instructs Cb precursors to adopt an early born cell fate.

Our analysis also indicates that early *Gbx2*-derived neurons are distributed within the two Cb germinal zones at E12.5. With early marking, a majority of r1 is derived from the *Gbx2* lineage, although a small cohort of unmarked cells is also observed. Over the course of development, these unmarked precursors apparently undergo massive proliferation to give rise to later born Cb cell types. In contrast, Cb precursors with a history of later *Gbx2* expression (E10.5 and E11.5) are not located in the most posterior VZ and URL at E12.5 (Figure 1). At these later embryonic stages, *Gbx2* expression is downregulated in the posterior Cb. Therefore, fate mapping at these stages only marks cells that have already emerged from the URL and posterior VZ by E12.5. By comparing all five fate mapping marking stages, we determined that early *Gbx2* expression does not necessarily translate into early migration away from the germinal zone. These findings establish that the timing of *Gbx2* expression does not regulate when the *Gbx2* lineage emerges from the VZ or URL.

Beyond birth order, the spatial distribution of the *Gbx2* lineage may also be attributed to morphogenic movements that establish the complex anatomy of the mature Cb. For example, the medial bias of *Gbx2*-derived granule cells may occur because the heavily labeled anterior folia are not contiguous with the Cb hemispheres (Altman and Bayer, 1997). In addition, the anterior-posterior axis of dorsal r1 undergoes a 90° rotation from E9.5 to E12.5 and is converted into the medial-lateral axis of the adult Cb (Sgaier et al., 2005). Therefore, any medial bias in labeling within the adult Cb may result from an anterior bias in *Gbx2* expression embryonically (Supplemental Figure 2). In particular, the medial distribution of *Gbx2*-derived granule cells and Purkinje cells at later marking stages is most likely explained by the down-regulation of Gbx2 expression in posterior r1 (See Figure 1). Moreover, the sharp decrease in granule cell marking from E8.5 to E11.5 and the relatively prolonged contribution to Purkinje cells suggests that *Gbx2* becomes restricted to the anterior ventricular zone as development proceeds.

The spatial biases in our GIFM analysis also provides new insights into the vermis phenotype of the conditional knock out of *Gbx2* at E8.5 (Li et al., 2002). Previous gene expression analysis revealed that the *Otx2* and *Fgf8* expression domains extend posteriorly into medial r1 in *Gbx2*-CKO mice. This posterior shift in gene expression verified that *Gbx2* continues to function in positioning and maintaining the IsO after E8.5 (Li et al., 2002). However, we show that the *Gbx2* lineage also preferentially contributes to cells located in the medial Cb. Specifically, the *Gbx2* lineage consistently gives rise to granule cells in the vermis across all fate mapping stages, while the contribution to the Cb hemispheres is significantly reduced after E9.5. These fate mapping results contribute to the explanation as to why the vermis is more affected by the loss of *Gbx2* expression after E8.5 and points to a cell autonomous role for *Gbx2* in Cb precursors.

The loss of *Gbx2* expression in *Gbx2*-CKO mice also allows the secreted glycoprotein, *Wnt1*, to be aberrantly expressed within r1—in addition to endogenous *Wnt1* expression in the rhombic lip (Hagan and Zervas, 2012). Interestingly, the expanded *Wnt1* expression domain is only observed medially and may help explain the vermis phenotype in *Gbx2*-CKO mice (Li et al., 2002). In *Gbx2*^−/−^ mice, *Wnt1* expression also extends into r1. Moreover, *Gbx2*^−/−^*;Otx2*^−/−^ double knockout mutants confirm that that the enlarged *Wnt1* expression domain is not purely because of a posterior shift in *Otx2* expression (Wassarman et al., 1997; Li and Joyner, 2001). Therefore, *Gbx2* likely functions to cell autonomously repress *Wnt1* expression in r1 precursors. Importantly, this repression not only occurs at the interface of the IsO and r1, but also delays the onset of *Wnt1* expression in the URL. Notably, at E7.5 and E8.5 the expression of *Gbx2* extends throughout r1 and *Wnt1* is not expressed in the Cb primordia. However, by E9.5 *Gbx2* starts to diminish particularly in posterior r1 and may allow for *Wnt1* expression to be upregulated in the upper rhombic lip at E10.5 (Wilkinson et al., 1987; Hagan and Zervas, 2012). In fact, GIFM experiments tracking the temporal contribution of the *Wnt1* lineage revealed that *Wnt1*-derived granule cells marked at later embryonic stages are generally distributed in a complementary pattern to the *Gbx2*-derived granule cells (this study and Hagan and Zervas, 2012). In this regard, the *Gbx2* lineage gives rise to the earliest progenitors emerging from the VZ and URL, while the *Wnt1* lineage produces progenitors arising from the URL slightly later in development (Hagan and Zervas, 2012). Together, these results suggest that *Gbx2* plays a cell autonomous role in regulating the timing of *Wnt1* expression in r1. Through this temporal control of gene expression, a combinatorial molecular code may emerge to determine the cell fate of precursors within the developing Cb.

The Cb has a prominent role in complex disorders including Tuberous Sclerosis and autism (Tsai et al., 2012; Hampson and Blatt, 2015; Mosconi et al., 2015; reviewed in D'Mello and Stoodley, 2015). The anatomical domains Crus I/II have emerged as being seminal in autism (D'Mello et al., 2015) and a reduction of Purkinje cells is a cellular correlate to autism phenotypes (Bauman and Kemper, 2005). Consistent gray matter reductions that occur in autism are in Crus I and lobules VIIIB, and IX (Stoodley, 2014). Interestingly, these regions have decreased *Gbx2* contribution beginning at E10.5 (Figure 5), which is nearly coincident with the peak of *Wnt1* contributation to these domains (Hagan and Zervas, 2012). We believe that our current study linking *Gbx2* expression, cell lineage, and cell fate in the Cb may provide valuable context for understanding neurological disease. Determining the temporal relationships between *Gbx2* expressing precursors and the cell types that this lineage gives rise to is a valuable guide for understanding the organization of the Cb. Additionally, these findings will be fruitful for interpreting when specific Cb domains, Cb cell types, and behaviors may be altered in human disease or in experimental mouse models of neurological disease.

## Ethics statement

Mice were housed and handled in accordance with Brown University Institutional Animal Care and Use Committee (IACUC) guidleines (Genetic Approaches Using Mus Musculus as a Model Organism to Understand Mechanisms Underpinning Neurodevelopment and Neurological Disorders *in Vivo*,” IACUC #1209030). This research was supported by startup funds (MZ).

## Author contributions

The original experimental approach was designed by MZ. Experiments were conducted by NH, JG, and DE. The manuscript was written and edited by MZ, NH, and JG. Research presented here fulfilled in part the Ph.D. thesis requirement for NH and the senior honors thesis for JG. ^*^NH and JG contributed equally to the manuscript.

### Conflict of interest statement

The authors declare that the research was conducted in the absence of any commercial or financial relationships that could be construed as a potential conflict of interest.

## Supplementary material

Supplemental Figure 1 GIFM strategy to mark and track the *Gbx2* lineage during Cb development. **(A)** Schematic of mouse embryo (right) showing cells containing both the *Gbx2*^*CreER*−*IRES*−*EGFP*/+^ and the *mGFP lox-STOP-lox-mGFP-IRES-NLS-LacZ-pA* reporter allele (left). CreER protein is sequestered in the cytoplasm by heat shock protein 90 (hsp90). **(B)** Tamoxifen (T) administration results in the release of CreER from hsp90 which is then free to enter the nucleus and engages the reporter allele, which is not initially expressed because of the *loxP* flanked *stop cassette* (red). **(C)** Once in the nucleus, CreER seeks out *loxP* sites (white triangles) and deletes the stop cassette which allows for expression of the GFP and nuclear LacZ reporter proteins. **(D)** Because *Gbx2* is expressed in r1 these cells with the reporter in the ON configuration are constitutively and heritably marked with high reproducibility. **(E)** In summary, the absence of tamoxifen, even in the presence of CreER and the reporter yields cells that are not marked. **(F)** However, the presence of CreER (driven by *Gbx2*) plus tamoxifen causes neurons to be marked with nuclear LacZ and mGFP.

Supplemental Figure 2 Summary schematic of the *Gbx2* lineage contribution to granule cells. The cerebellar primordium is located within the roster Hb (r1) and at E8.5 is partitioned into anterior r1 (rostral, indicated by the light green box) and posterior r1 (caudal, indicated by the light blue box). These domains are transposed 90 degrees to become the medial vermis and lateral hemispheres, respectively, of the adult Cb (based on Sgaier et al., 2005). A more nuanced description is that rostral-medial (RM) domains become posterior vermis (VP) while rostral-lateral (RL) domains become anterior vermis (VA). In contrast, the caudal-medial (CM) domains become posterior-hemisphere (HP) tissue and the caudal-lateral (CL) domains become anterior-hemisphere (HA) tissue. The density of stippling indicates the relative contribution to granule cells. The *Gbx2* lineage was marked at early (E7.5 and E8.5, orange), intermediate (E9.5, light blue), and late (E10.5 and E11.5, purple) embryonic time points and the distribution of *Gbx2*-derived granule cells

Supplemental Figure 3 Summary schematic of the *Gbx2* lineage contribution to Purkinje cells. The cerebellar primordium is located within the roster Hb (r1) and at E8.5 is partitioned into anterior r1 (rostral, indicated by the light green box) and posterior r1 (caudal, indicated by the light blue box). These domains are transposed 90 degrees to become the medial vermis and lateral hemispheres, respectively, of the adult Cb (based on Sgaier et al., 2005). Specifically, rostral-medial (RM) domains become posterior vermis (VP) while rostral-lateral (RL) domains become anterior vermis (VA). In contrast, the caudal-medial (CM) domains become posterior-hemisphere (HP) tissue and the caudal-lateral (CL) domains become anterior-hemisphere (HA) tissue. The density of stippling indicates the relative contribution to Purkinje cells. The *Gbx2* lineage was marked at early (E7.5 and E8.5, orange), intermediate (E9.5, light blue), and late (E10.5 and E11.5, purple) embryonic time points and the distribution of *Gbx2*-derived granule cells plotted.

## References

[B1] Altman J. Bayer S. (1997). Development of the Cerebellar System in Relation to Its Evolution, Structure, and Function. Boca Raton, FL: CRC Press.

[B2] Bastianelli E. (2003). Distribution of calcium-binding proteins in the cerebellum. Cerebellum 2, 242–262. 10.1080/14734220310022289 14964684

[B3] Bauman M. L. Kemper T. L. (2005). Neuroanatomic observations of the brain in autism: a review and future directions. Int. J. Dev. Neurosci. 23, 183–187. 10.1016/j.ijdevneu.2004.09.006 15749244

[B4] Brown A. Brown S. Ellisor D. Hagan N. Normand E. Zervas M. (2009). A practical approach to genetic inducible fate mapping: a visual guide to mark and track cells *in vivo*. J. Vis. Exp. 43:1687 10.3791/1687 PMC2846818 20042997

[B5] Buckner R. L. (2013). The cerebellum and cognitive function: 25 years of insight from anatomy and neuroimaging. Neuron 80, 807–815. 10.1016/j.neuron.2013.10.044 24183029

[B6] Chen L. Guo Q. Li J. Y. (2009). Transcription factor Gbx2 acts cell-nonautonomously to regulate the formation of lineage-restriction boundaries of the thalamus. Development 136, 1317–1326. 10.1242/dev.030510 19279136 PMC2687463

[B7] D'Mello A. M. Crocetti D. Mostofsky S. H. Stoodley C. J. (2015). Cerebellar gray matter and lobular volumes correlate with core autism symptoms. Neuroimage Clin. 7, 631–639. 10.1016/j.nicl.2015.02.007 25844317 PMC4375648

[B8] D'Mello A. M. Stoodley C. J. (2015). Cerebro-cerebellar circuits in autism spectrum disorder. Front. Neurosci. 9:408. 10.3389/fnins.2015.00408 26594140 PMC4633503

[B9] Dymecki S. M. Kim J. C. (2007). Molecular neuroanatomy's “Three Gs”: a primer. Neuron 54, 17–34. 10.1016/j.neuron.2007.03.009 17408575 PMC2897592

[B10] Ellisor D. Koveal D. Hagan N. Brown A. Zervas M. (2009). Comparative analysis of conditional reporter alleles in the developing embryo and embryonic nervous system. Gene Expr. Patterns 9, 475–489. 10.1016/j.gep.2009.07.007 19616131 PMC2855890

[B11] Ellisor D. Zervas M. (2010). Tamoxifen dose response and conditional cell marking: is there control? Mol. Cell. Neurosci. 45, 132–138. 10.1016/j.mcn.2010.06.004 20600933

[B12] Englund C. Kowalczyk T. Daza R. A. M. Dagan A. Lau C. Rose M. F. Hevner R. F. (2006). Unipolar brush cells of the cerebellum are produced in the rhombic lip and migrate through developing white matter. J. Neurosci. 26, 9184–9195. 10.1523/JNEUROSCI.1610-06.2006 16957075 PMC6674506

[B13] Hagan N. Zervas M. (2012). Wnt1 expression temporally allocates upper rhombic lip progenitors and defines their terminal cell fate in the cerebellum. Mol. Cell. Neurosci. 49, 217–229. 10.1016/j.mcn.2011.11.008 22173107 PMC3351839

[B14] Hampson D. R. Blatt G. J. (2015). Autism spectrum disorders and neuropathology of the cerebellum. Front. Neurosci. 9:420 10.3389/fnins.2015.00420 26594141 PMC4635214

[B15] Hippenmeyer S. Vrieseling E. Sigrist M. Portmann T. Laengle C. Ladle D. R. (2005). A developmental switch in the response of DRG neurons to ETS transcription factor signaling. PLoS Biol. 3:e159 10.1371/journal.pbio.0030159 15836427 PMC1084331

[B16] Hoshino M. Nakamura S. Mori K. Kawauchi T. Terao M. Nishimura Y. V.. (2005). Ptf1a, a bHLH transcriptional gene, defines GABAergic neuronal fates in cerebellum. Neuron 47, 201–213. 10.1016/j.neuron.2005.06.007 16039563

[B17] Joyner A. L. Zervas M. (2006). Genetic inducible fate mapping in mouse: establishing genetic lineages and defining genetic neuroanatomy in the nervous system. Dev. Dyn. 235, 2376–2385. 10.1002/dvdy.20884 16871622

[B18] Leto K. Carletti B. Williams I. M. Magrassi L. Rossi F. (2006). Different types of cerebellar GABAergic interneurons originate from a common pool of multipotent progenitor cells. J. Neurosci. 26, 11682–11694. 10.1523/JNEUROSCI.3656-06.2006 17093090 PMC6674781

[B19] Li J. Y. Joyner A. L. (2001). Otx2 and Gbx2 are required for refinement and not induction of mid- hindbrain gene expression. Development 128, 4979–4991. 11748135 10.1242/dev.128.24.4979

[B20] Li J. Y. Lao Z. Joyner A. L. (2002). Changing requirements for Gbx2 in development of the cerebellum and maintenance of the mid/hindbrain organizer. Neuron 36, 31–43. 10.1016/S0896-6273(02)00935-2 12367504

[B21] Liu A. Joyner A. L. (2001). Early anterior/posterior patterning of the midbrain and cerebellum. Annu. Rev. Neurosci. 24, 869–896. 10.1146/annurev.neuro.24.1.869 11520921

[B22] Luu B. Ellisor D. Zervas M. (2011). The lineage contribution and role of Gbx2 in spinal cord development. PLoS ONE 6:e20940. 10.1371/journal.pone.0020940 21698205 PMC3116860

[B23] Machold R. Fishell G. (2005). Math1 is expressed in temporally discrete pools of cerebellar rhombic-lip neural progenitors. Neuron 48, 17–24. 10.1016/j.neuron.2005.08.028 16202705

[B24] Madisen L. Zwingman T. A. Sunkin S. M. Oh S. W. Zariwala H. A. Gu H. (2010). A robust and high-throughput Cre reporting and characterization system for the whole mouse brain. Nat. Neurosci. 13, 133–140. 10.1038/nn.2467 20023653 PMC2840225

[B25] Maricich S. M. Herrup K. (1999). Pax-2 expression defines a subset of GABAergic interneurons and their precursors in the developing murine cerebellum. J. Neurobiol. 41, 281–294. 10.1002/(SICI)1097-4695(19991105)41:2<281::AID-NEU10>3.0.CO;2-5 10512984

[B26] Millet S. Campbell K. Epstein D. J. Losos K. Harris E. Joyner A. L. (1999). A role for Gbx2 in repression of Otx2 and positioning the mid/hindbrain organizer. Nature 401, 161–164. 10.1038/43664 10490024

[B27] Mosconi M. W. Wang Z. Schmit L. M. Tsai P. Sweeney J. A. (2015). The role of cerebellar circuitry alterations in the pathophysiology of autism spectrum disorders. Front. Neurosci. 9:296. 10.3389/fnins.2015.00296 26388713 PMC4555040

[B28] Reith R. M. McKenna J. Wu H. Hashmi S. S. Cho S. H. Dash P. K.. (2013). Loss of Tsc2 in Purkinje cells is associated with autistic-like behavior in a mouse model of tuberous sclerosis complex. Neurobiol. Dis. 51, 93–103. 10.1016/j.nbd.2012.10.014 23123587

[B29] Sato T. Joyner A. L. (2009). The duration of Fgf8 isthmic organizer expression is key to patterning different tectal-isthmo-cerebellum structures. Development 136, 3617–3626. 10.1242/dev.041210 19793884 PMC2761110

[B30] Sgaier S. K. Millet S. Villanueva M. P. Berenshteyn F. Song C. Joyner A. L. (2005). Morphogenetic and cellular movements that shape the mouse cerebellum; insights from genetic fate mapping. Neuron 45, 27–40. 10.1016/j.neuron.2004.12.021 15629700

[B31] Sillitoe R. V. Joyner A. L. (2007). Morphology, molecular codes, and circuitry produce the three- dimensional complexity of the cerebellum. Annu. Rev. Cell Dev. Biol. 23, 549–577. 10.1146/annurev.cellbio.23.090506.123237 17506688

[B32] Stoodley C. J. (2014). Distinct regions of the cerebellum show gray matter decreases in autism, ADHD, and developmental dyslexia. Front. Syst. Neurosci. 8:92. 10.3389/fnsys.2014.00092 24904314 PMC4033133

[B33] Ten Donkelaar H. J. Lammens M. (2009). Development of the human cerebellum and its disorders. Clin. Perinatol. 3, 513–530. 10.1016/j.clp.2009.06.001 19732611

[B34] Tsai P. T. Hull C. Chu Y. Greene-Colozzi E. Sadowski A. R. Leech J. M.. (2012). Autistic-like behaviour and cerebellar dysfunction in Purkinje cell Tsc1 mutant mice. Nature 488, 647–651. 10.1038/nature11310 22763451 PMC3615424

[B35] Wagner M. J. Kim T. H. Savall J. Schnitzer M. J. Luo L. (2017). Cerebellar granule cells encode the expectation of reward. Nature 544, 96–100. 10.1038/nature21726 28321129 PMC5532014

[B36] Wang V. Y. Rose M. F. Zoghbi H. Y. (2005). Math1 expression redefines the rhombic lip derivatives and reveals novel lineages within the brainstem and cerebellum. Neuron 48, 31–43. 10.1016/j.neuron.2005.08.024 16202707

[B37] Wassarman K. M. Lewandoski M. Campbell K. Joyner A. L. Rubenstein J. L. Martinez S.. (1997). Specification of the anterior hindbrain and establishment of a normal mid/hindbrain organizer is dependent on Gbx2 gene function. Development 124, 2923–2934. 9247335 10.1242/dev.124.15.2923

[B38] Wilkinson D. G. Bailes J. A. McMahon A. P. (1987). Expression of the proto-oncogene int-1 is restricted to specific neural cells in the developing mouse embryo. Cell 50, 79–88. 10.1016/0092-8674(87)90664-7 3594565

[B39] Zervas M. Blaess S. Joyner A. L. (2005). Classical embryological studies and modern genetic analysis of midbrain and cerebellum development. Curr. Top. Dev. Biol. 69, 101–138. 10.1016/S0070-2153(05)69005-9 16243598

[B40] Zervas M. Millet S. Ahn S. Joyner A. L. (2004). Cell behaviors and genetic lineages of the mesencephalon and rhombomere 1. Neuron 43, 345–357. 10.1016/j.neuron.2004.07.010 15294143

