# Supplementary figures and images for: The Temporal Contribution of the Gbx2 Lineage to Cerebellar Neurons
Source: Front Neuroanat. 2017 Jul 21;11:50. doi: 10.3389/fnana.2017.00050 (PMC5519623; doi:10.3389/fnana.2017.00050)

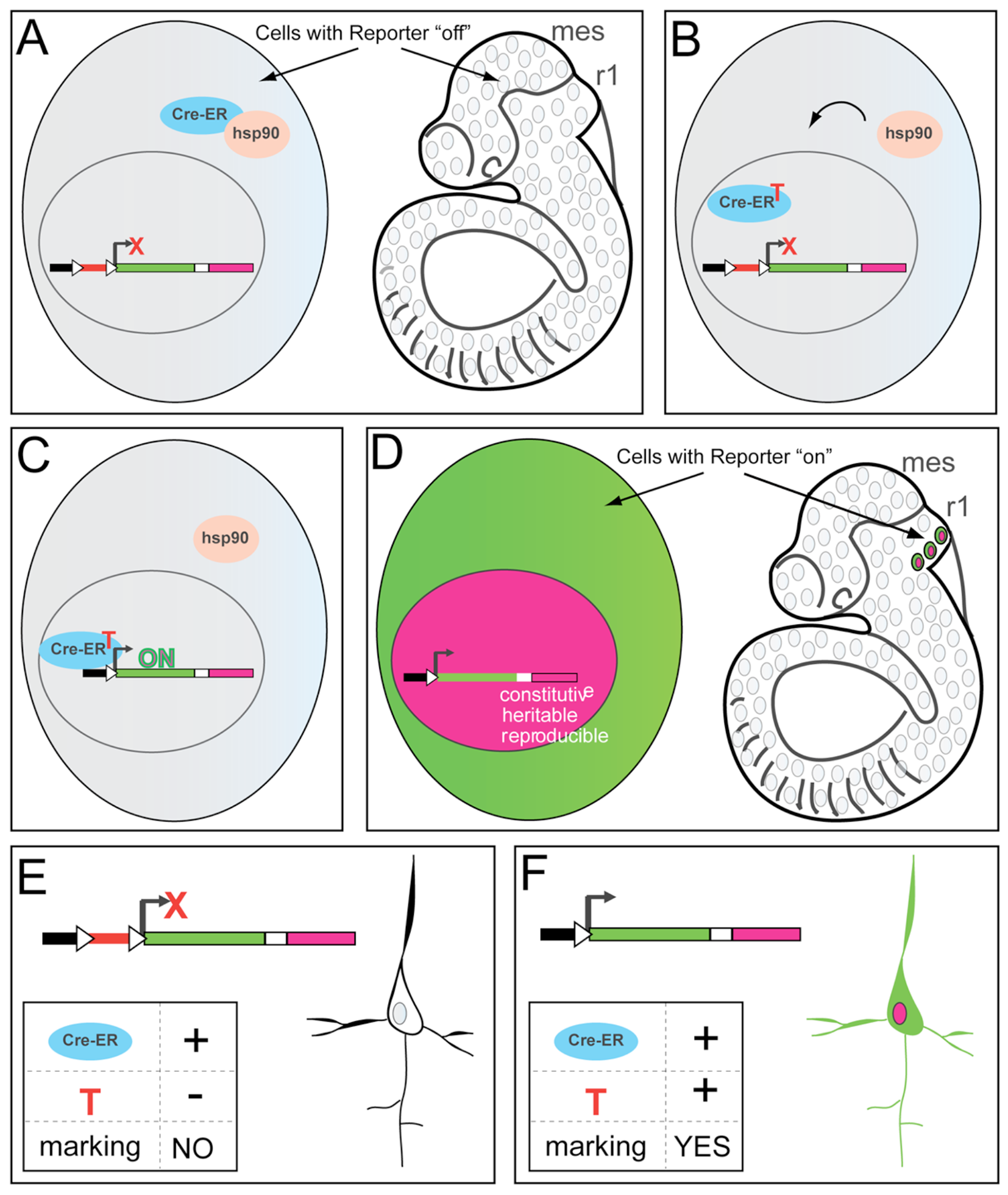

Supplement: Supplemental Figure 1 — GIFM strategy to mark and track the Gbx2 lineage during Cb development. (A) Schematic of mouse embryo (right) showing cells containing both the Gbx2CreER−IRES−EGFP/+ and the mGFP lox-STOP-lox-mGFP-IRES-NLS-LacZ-pA reporter allele (left). CreER protein is sequestered in the cytoplasm by heat shock protein 90 (hsp90). (B) Tamoxifen (T) administration results in the release of CreER from hsp90 which is then free to enter the nucleus and engages the reporter allele, which is not initially expressed because of the loxP flanked stop cassette (red). (C) Once in the nucleus, CreER seeks out loxP sites (white triangles) and deletes the stop cassette which allows for expression of the GFP and nuclear LacZ reporter proteins. (D) Because Gbx2 is expressed in r1 these cells with the reporter in the ON configuration are constitutively and heritably marked with high reproducibility. (E) In summary, the absence of tamoxifen, even in the presence of CreER and the reporter yields cells that are not marked. (F) However, the presence of CreER (driven by Gbx2) plus tamoxifen causes neurons to be marked with nuclear LacZ and mGFP. [file Image1.TIF]

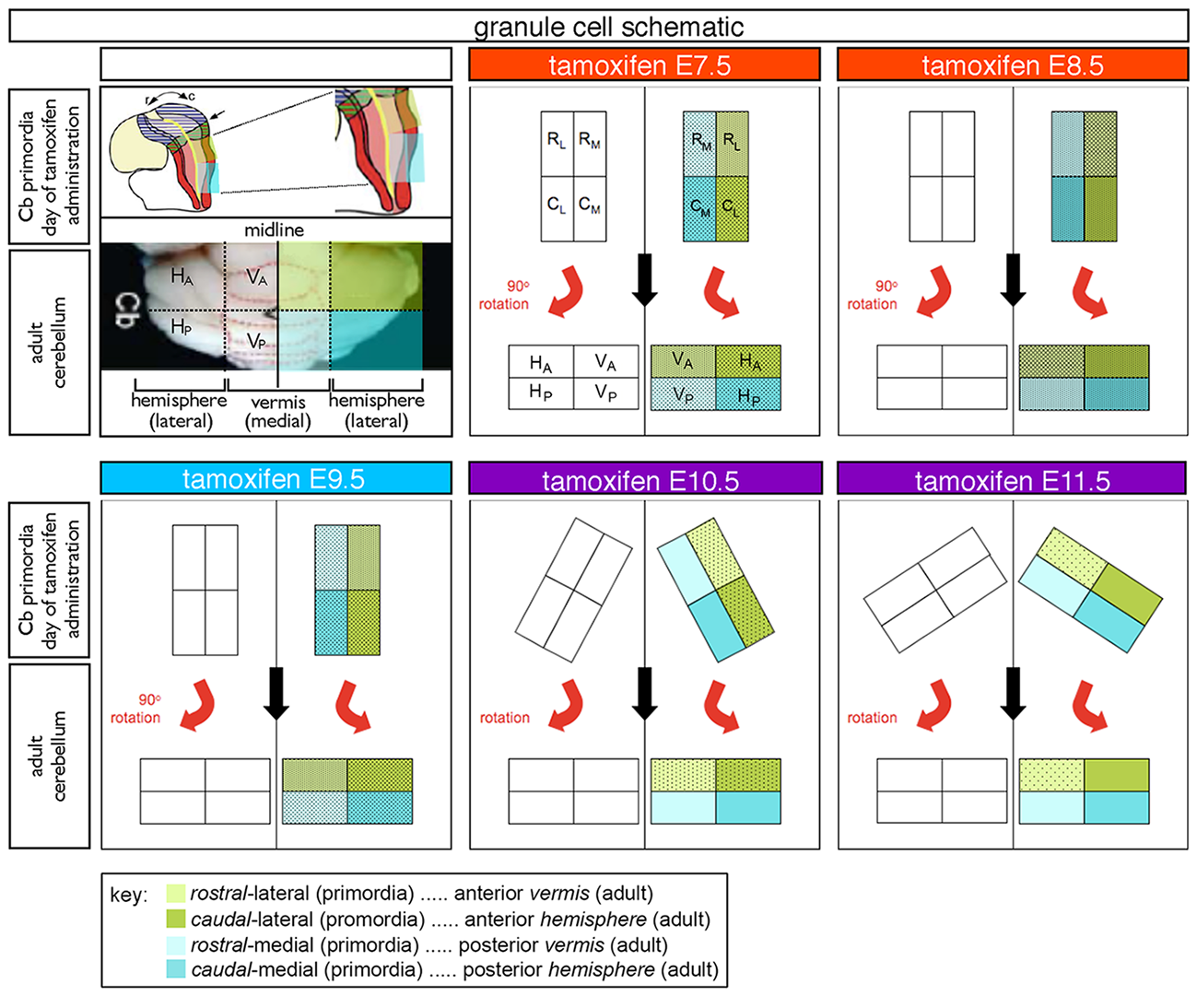

Supplement: Supplemental Figure 2 — Summary schematic of the Gbx2 lineage contribution to granule cells. The cerebellar primordium is located within the roster Hb (r1) and at E8.5 is partitioned into anterior r1 (rostral, indicated by the light green box) and posterior r1 (caudal, indicated by the light blue box). These domains are transposed 90 degrees to become the medial vermis and lateral hemispheres, respectively, of the adult Cb (based on Sgaier et al., 2005). A more nuanced description is that rostral-medial (RM) domains become posterior vermis (VP) while rostral-lateral (RL) domains become anterior vermis (VA). In contrast, the caudal-medial (CM) domains become posterior-hemisphere (HP) tissue and the caudal-lateral (CL) domains become anterior-hemisphere (HA) tissue. The density of stippling indicates the relative contribution to granule cells. The Gbx2 lineage was marked at early (E7.5 and E8.5, orange), intermediate (E9.5, light blue), and late (E10.5 and E11.5, purple) embryonic time points and the distribution of Gbx2-derived granule cells [file Image2.tif]

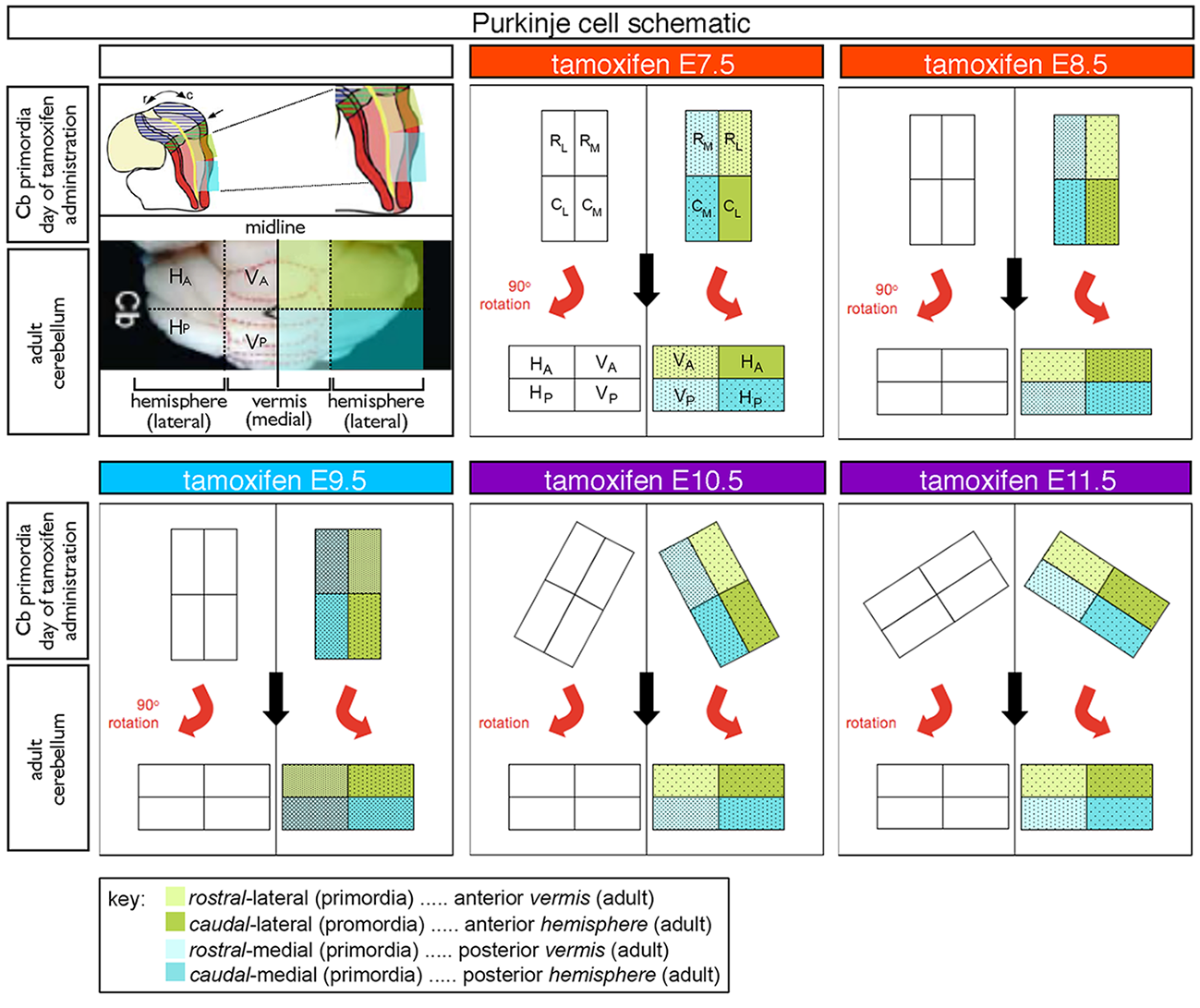

Supplement: Supplemental Figure 3 — Summary schematic of the Gbx2 lineage contribution to Purkinje cells. The cerebellar primordium is located within the roster Hb (r1) and at E8.5 is partitioned into anterior r1 (rostral, indicated by the light green box) and posterior r1 (caudal, indicated by the light blue box). These domains are transposed 90 degrees to become the medial vermis and lateral hemispheres, respectively, of the adult Cb (based on Sgaier et al., 2005). Specifically, rostral-medial (RM) domains become posterior vermis (VP) while rostral-lateral (RL) domains become anterior vermis (VA). In contrast, the caudal-medial (CM) domains become posterior-hemisphere (HP) tissue and the caudal-lateral (CL) domains become anterior-hemisphere (HA) tissue. The density of stippling indicates the relative contribution to Purkinje cells. The Gbx2 lineage was marked at early (E7.5 and E8.5, orange), intermediate (E9.5, light blue), and late (E10.5 and E11.5, purple) embryonic time points and the distribution of Gbx2-derived granule cells plotted. [file Image3.tif]
